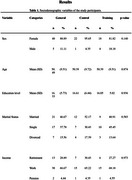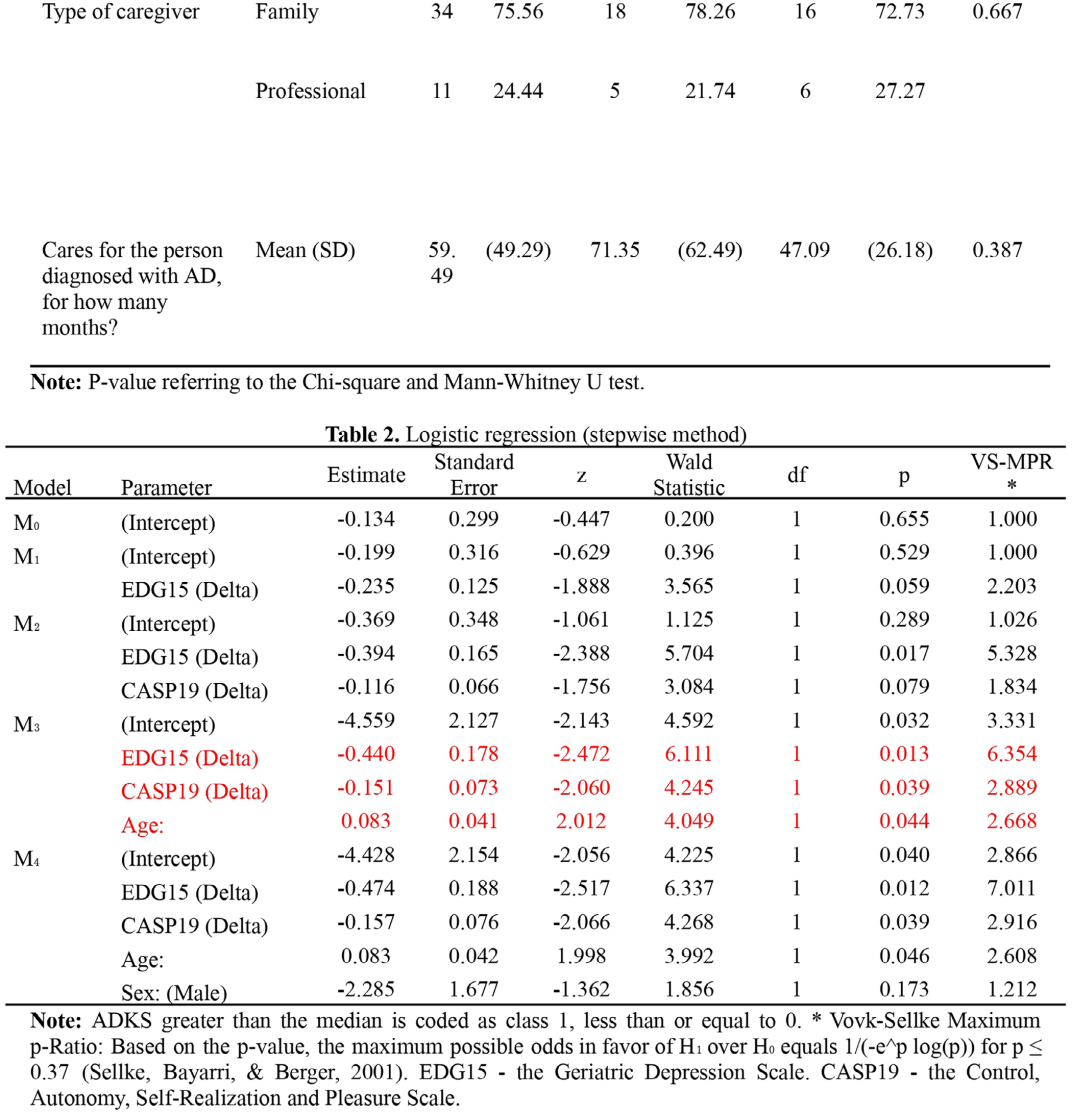# Psychoeducation focusing on cognitive stimulation for caregivers of elderly people diagnosed with Alzheimer's Disease: A randomized controlled intervention study

**DOI:** 10.1002/alz70858_102168

**Published:** 2025-12-25

**Authors:** Ana Paula Bagli Moreira, Tiago Nascimento Ordonez, Beatriz Aparecida Ozello Gutierrez, Thais Bento Lima Silva

**Affiliations:** ^1^ Gerontology of the School of Arts, Science and Humanities of the University of São Paulo, São Paulo, São Paulo, Brazil; ^2^ Federal University of Recôncavo da Bahia, Santo Antônio de Jesus, Bahia, Brazil; ^3^ Cognitive and Behavioral Neurology Group of the University of São Paulo School of Medicine, São Paulo, São Paulo, Brazil

## Abstract

**Background:**

The literature reports that caregivers of older people with dementia face major challenges concerning mental health and wellbeing. Hence, assistance for caregivers should be prioritized in public policies on aging.

**Method:**

A total of 45 caregivers took part, randomly stratified by age and education into 2 groups: a Training Group (TG) with 22 participants; and a Control Group (CG) with 23 participants. The Training Group had 12 meetings of 1.5 hours with members of the multi‐disciplinary dementia team, After the series of meetings had finished, participants engaged in cognitive group activities remotely via the Google Meet platform conducted by the researchers. Subsequently, participants were advised to engage in cognitive activities on the Supera Online Platform for 30 minutes per week. Protocol: the sociodemographic and health questionnaire, the Alzheimer´s Disease Knowledge Scale (ADKS), the Mini‐Mental State Exam for telephone application (MEEM‐Braztel), the Subjective Cognitive Decline Questionnaire (SCD‐Q), the Geriatric Depression Scale (GDS‐15), the Geriatric Anxiety Inventory (GAI), the Depression, Anxiety and Stress Scale (DASS‐21), the Caregiver Burden Scale (ZARIT), the Control, Autonomy, Self‐Realization and Pleasure (CASP‐19) scale, questionnaire on positive and negative mood states, and general Satisfaction with life domains scale.

**Result:**

The caregivers in the TG had positive impacts for some aspects after participating in the intervention, such as for the perceived quality of life variable, as measured by the CASP‐19, the CG showed a decrease of ‐3.52 and the TG an increase of 1.86 (*p* = 0.014). On the ADKS for the domains “Treatment and Management” (*p* = 0.005) and “Course” (*p* = 0.056), there was an increase in *p*‐value (*p* = 0.029) versus time, although this was not significant for Time x Group (*p* = 0.686) on the ADKS.

**Conclusion:**

The study found that caregivers who participated in the 12 intervention sessions held greater knowledge on Alzheimer´s Disease, showing better management for dealing with dementia‐related situations. Also, the psychological and sociodemographic aspects, such as less depressive symptoms, better perceived quality of life, and age, were positive predictors for holding greater knowledge on Alzheimer´s Disease.